# A method utilizing chemical carcinogens and a Western diet to reliably induce advanced hepatocellular carcinoma in male and female mice

**DOI:** 10.1186/s40170-026-00441-6

**Published:** 2026-06-09

**Authors:** Melina C. Mancini, Elise R. Adams, David H. Burk, Richard P. Carmouche, Sara M. Webb, Jaroslaw Staszkiewicz, J. Michael Salbaum, Samuel G. Mackintosh, Timothy D. Heden

**Affiliations:** 1 Molecular Metabolism Lab, Pennington Biomedical Research Center, Baton Rouge, LA 70808 USA; 2 Cell Biology and Bioimaging Core, Pennington Biomedical Research Center, Baton Rouge, LA USA; 3 Genomics Core, Pennington Biomedical Research Center, Baton Rouge, LA USA; 4 Molecular Mechanisms Core, Pennington Biomedical Research Center, Baton Rouge, LA USA; 5 Department of Biochemistry and Molecular Biology, University of Arkansas for Medical Sciences, Little Rock, AR USA

**Keywords:** Hepatocellular carcinoma, Chemical method to induce liver cancer, Liver spatial transcriptomics, Proteomics, Diethylnitrosamine, Thioacetamide, Sucrose

## Abstract

**Background:**

A combination of chemical carcinogens and Western diet have been used to induce hepatocellular carcinoma (HCC) in mice, but these models show low incidence of HCC in female mice, while mice that do develop HCC show a wide range of HCC stage development. Therefore, a more reliable method that induces advanced stage HCC in both male and female mice is warranted. The purpose of this study was to create a simple, yet tolerable and reliable method that utilizes chemical carcinogens and a Western diet to induce advanced stage HCC in both male and female mice.

**Methods:**

Mice were provided with a low-fat control diet or Western diet at weaning and for the remainder of their 30-week lifetime. To induce HCC, mice given the Western diet received the sequential administration of low dose diethylnitrosamine (DEN), thioacetamide (TAA), and sucrose water.

**Results:**

This method produced stage 2–3 HCC by 30 weeks of age in 100% of male mice and 96% of female mice (4% had stage 1 HCC), with a low fatality rate. Spatial transcriptomics, proteomics, and western blotting revealed this method alters gene and protein markers of HCC in a similar fashion compared to human HCC.

**Conclusions:**

This study introduces a new method that utilizes chemical carcinogens and a Western diet to reliably induce advanced HCC in both male and female mice. Importantly, the method was tolerable while the HCC model is translational as it induced protein and gene signatures comparable to human advanced HCC. This new method provides a valuable HCC model that can be used to better understand the pathogenesis of HCC or for preclinical testing of new therapeutic approaches to treat advanced HCC.

**Clinical trial number:**

Not applicable.

**Supplementary Information:**

The online version contains supplementary material available at https://doi.org/10.1186/s40170-026-00441-6.

## Background

Hepatocellular carcinoma (HCC) is the most common liver malignancy (> 90% of cases) [1] and the fifth most common cancer worldwide [2]. More than 800,000 people are diagnosed with HCC worldwide each year [1]. The five-year survival of untreated HCC is 9.6% [3]. While treatments such as liver transplantation, tumor ablation, open resection, minimally-invasive resection, transarterial (chemo)embolization, or selective internal radiation therapy can improve five-year survival rates, the majority of HCC patients do not have access to these therapies [3]. Thus, more research is needed to help develop more practical and far-reaching treatment strategies for HCC.

The development of treatments begins with efficacy studies in preclinical models. In mice, diethylnitrosamine (DEN) is a common genotoxic carcinogen given to promote HCC initiation. DEN reacts with cytochrome P450 enzymes in the liver to form alkylating metabolites that alkylate deoxyribonucleic acid (DNA), forming mutagenic DNA adducts that promote HCC. The traditional approach includes administering a single dose of DEN via an intraperitoneal injection to young mice at two weeks of age. When mice are fed standard chow diets, this approach is well tolerated, but it can take 10 months (~ 43 weeks) or longer to promote HCC [4–9]. Moreover, there is an uneven occurrence time with some reports indicating that not all mice develop HCC [6, 7, 9, 10], with female mice having a much lower incidence compared to male mice [11–14]. Combining DEN with other chemotoxic agents such as thioacetamide (TAA), carbon tetrachloride, ethanol, lipopolysaccharide, and/or a Western diet [9, 15–18] has been shown to increase the occurrence and speed at which HCC develops, but female mice still have less HCC occurrence, with some mice not developing HCC even at 40 weeks of age [16]. Moreover, given the fact that HCC is most often diagnosed in humans at an advanced stage [3], a protocol is warranted that can consistently induce advanced stage HCC in both male and female mice, which would be a useful tool to not only study the pathogenesis of HCC but allow the reliable testing of new therapeutics.

Here we report a tolerable and reliable method to induce advanced stage 2–3 HCC [19] in both male and female mice. The method involves providing mice with a Western diet at weaning and for their lifetime combined with multiple low dose DEN injections starting at 2 weeks of age for eight weeks, followed by TAA drinking water for four weeks, and 10% sucrose water for the remainder of their life. At 30 weeks of age 100% of male mice and 96% of female mice develop advanced stage 2–3 HCC with large visible tumors that have a gene and protein expression pattern comparable to what has been observed in human HCC.

## Methods

### Mouse model

**Fig. 1:**
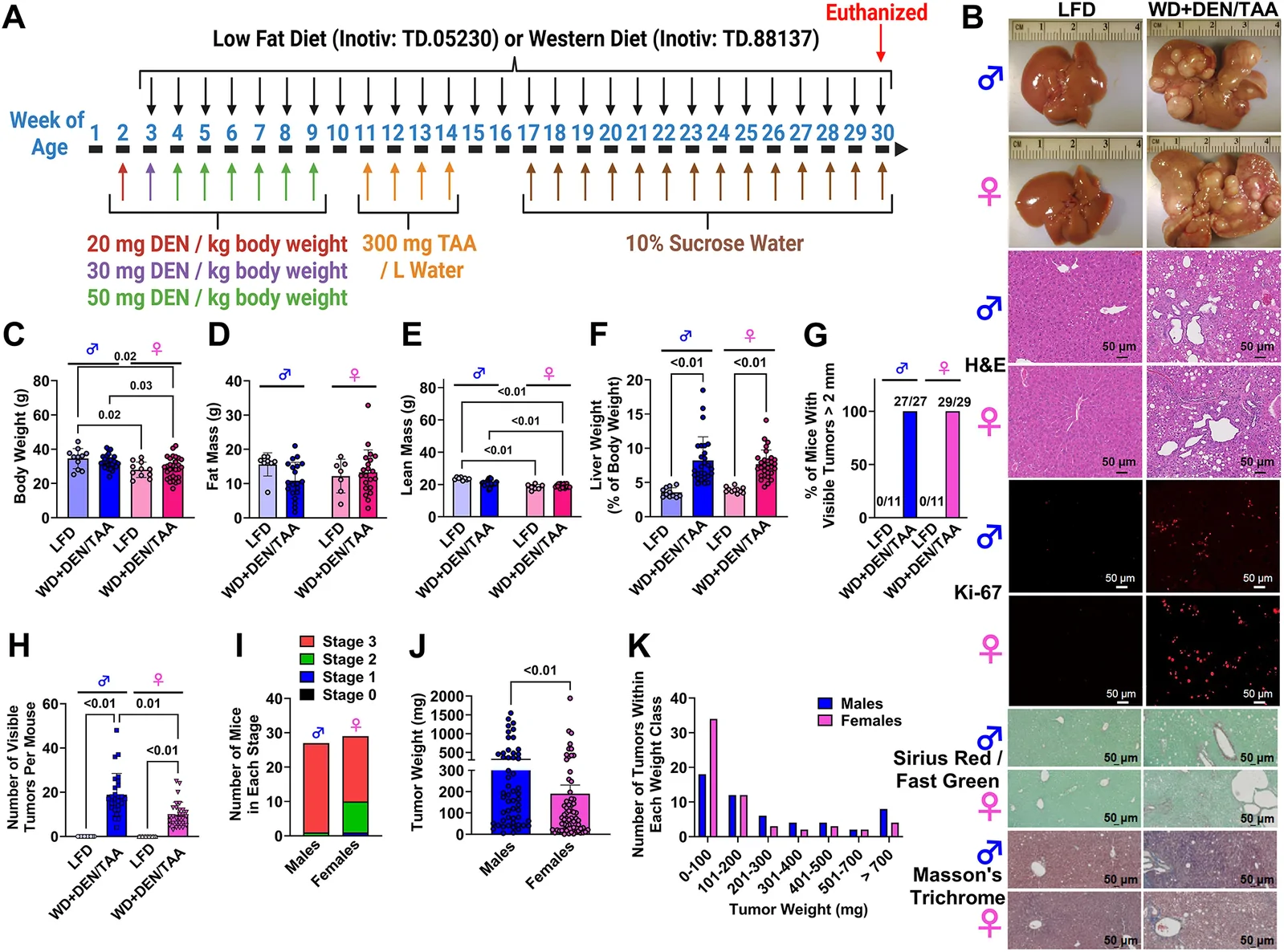
A method to reliably produce advanced stage 2–3 HCC in male and female mice. **(A)** Illustration showing details of method to induce advanced HCC in male and female mice. The method involved the sequential administration of DEN, TAA, and 10% sucrose water, all on top of a Western diet. **(B)** Representative image of whole liver (top), hematoxylin and eosin staining (H&E) (second from top), Ki-67 staining (third from top), Sirius Red / Fast Green staining (fourth from top), and Masson’s Trichrome (bottom) staining of liver sections from male and female mice. **(C)** Final body weight of the mice (*n* = 11 LFD fed males, *n* = 27 WD + DEN/TAA males, *n* = 11 LFD fed females, and *n* = 29 WD + DEN/TAA females). **(D)** NMR measured fat mass (*n* = 8 LFD fed males, *n* = 20 WD + DEN/TAA males, *n* = 7 LFD fed females, and *n* = 21 WD + DEN/TAA females). **(E)** NMR measured lean mass (*n* = 8 LFD fed males, *n* = 20 WD + DEN/TAA males, *n* = 7 LFD fed females, and *n* = 21 WD + DEN/TAA females). **(F)** Liver weight, normalized to body weight (*n* = 11 LFD fed males, *n* = 27 WD + DEN/TAA males, *n* = 11 LFD fed females, and *n* = 29 WD + DEN/TAA females. **(G)** Percentage of mice with visible tumors > 2 mm in diameter. **(H)** The total number of visible tumors per mouse (*n* = 11 LFD fed males, *n* = 27 WD + DEN/TAA males, *n* = 11 LFD fed females, and *n* = 29 WD + DEN/TAA females). **(I)** Number of mice with stage 0, stage 1, stage 2, or stage 3 HCC (*n* = 27 WD + DEN/TAA males, *n* = 29 WD + DEN/TAA females. **(J)** Tumor weights (*n* = 54 male tumors, *n* = 62 female tumors. A total of 2–3 tumors per mouse were weighed and included in the data (*n* = 54 WD + DEN/TAA male tumors, *n* = 62 WD + DEN/TAA female tumors). **(K)** Frequency distribution of a range of tumor weights (*n* = 54 WD + DEN/TAA males, *n* = 62 WD + DEN/TAA females). Where applicable, data are presented as means ± S.D. Data were analyzed using two-way ANOVA’s with a follow-up Tukey’s multiple comparisons test. Figure 1**J** was analyzed using an unpaired t-tests

This research was conducted in accordance with both the Declarations of Helsinki and Istanbul. This study was approved by the Pennington Biomedical Research Center’s Institutional Animal Care and Use Committee. A total of 78 female and male mice were used for this study. The mice had a mixed background including C57BL/6J and C57BL/6N. All animals were group housed with a 12-hour light and 12-hour dark cycle (light between 7 am to 7 pm) at room temperatures ranging between ~ 22–23 °C. Two groups of mice were used: (1) a low-fat diet (LFD) control group (TD.05230, 18.7% protein, 68.7% carbohydrate, 12.6% fat, Inotiv, West Lafayette, IN, USA) with 11 males and 11 females, and (2) a Western diet (WD) fed group (TD.88137, 15.2% protein, 42.7% carbohydrate (34% sucrose), 42% fat, Inotiv) with 27 males and 29 females that received the chemical carcinogens diethylnitrosamine (DEN, D0516, TCI America, Montgomeryville, PA, USA) and thioacetamide (TAA, 172502, Millipore Sigma, Saint Louis, MO, USA) (WD + DEN/TAA). Mice were placed on their respective diet after weaning. Mice in the WD + DEN/TAA group began receiving intraperitoneal injections of DEN once a week for eight weeks starting at two weeks of age (Fig. 1A). This included 20 mg of DEN per kg of body weight for the first week, 30 mg of DEN per kg of body weight the second week, and 50 mg of DEN per kg of body weight for the next six weeks. This protocol was modified from previous reports [20, 21]. One week after completion of DEN injections, mice were provided with TAA drinking water (300 mg/L) for a period of four weeks straight to promote liver damage, liver fibrosis, and accelerate HCC development. After two weeks of regular water, mice received 10% sucrose water to further promote HCC [9, 16], and the sucrose water was provided until euthanasia at 30 weeks of age. Two mice (out of 56 mice in WD + DEN/TAA, 3.5%) in the cancer group were euthanized prior to 30 weeks of age due to poor physical condition, indicating that the protocol is highly tolerable.

Prior to euthanasia, mice were anesthetized with a cocktail of ketamine (120 mg per kg of body weight), xylazine (9 mg per kg of body weight), and acepromazine (2 mg per kg of body weight) in saline via intraperitoneal injection. Once deep anesthesia was reached, blood was collected through a cardiac blood draw. Tissues were harvested after cervical dislocation. Tissues and serum were stored at − 80 °C.

### Liver imaging

Once the liver was removed, a picture of the liver was taken using a digital microscope camera (ODM701, Opqpq, China), the liver was weighed, adjacent, non-tumor tissues were collected, the total number of visible tumors were counted, and 2-3 tumors > 2 mm per mouse were dissected, weighed, and snap frozen in liquid nitrogen. The macroscopic liver nodules were used to assign the HCC stage as follows: stage 0 = no visible tumors; stage 1 = one to three nodules with diameter > 2 mm; stage 2 = four to six nodules > 2 mm, and stage 3 = seven or more nodules measuring > 2 mm [19]. Sections of liver tissue were preserved in 10% formalin overnight and then transferred to 70% ethanol prior to embedding and sectioning. Hematoxylin and Eosin (H & E) staining, Sirius Red / Fast Green staining, and Masson’s Trichrome staining were performed on liver sections within the Cell Biology and Bioimaging Subcore of the Molecular Mechanisms Core at Pennington Biomedical Research Center. Standard approaches were used for staining, as previously described [21–25]. Ki-67 staining was performed using the BOND RX Fully automated research stainer (Deer Park, IL, USA) with casein blocking solution (37583, Thermo Fisher Scientific, Waltham, MA, USA), the primary Ki-67 antibody at a concentration of 1:300 (ab15580, abcam, Cambridge, UK), and a goat anti-rabbit Alexa Fluor 647 secondary antibody (A32733, Invitrogen, Waltham, MA, USA) at a 1:300 dilution as previously described by our lab [21]. After staining, all slides were scanned using a Zeiss Axioscan 7 slide scanner (Carl Zeiss, Oberkochen, Germany).

### Body weight and composition

The body weight of the mice was measured using a digital pet scale for small animals (Mindpet-med, China). The body composition of the mice was measured using a Minispec LF90 Series TD-NMR System (Bruker Minispec LF90, Bruker, Karlsruhe, Germany).

### 10X visium spatial transcriptomics

Spatial transcriptomics was performed within both the Cell Biology and Bioimaging Core and the Genomics Core at Pennington Biomedical Research Center. Formalin fixed paraffin embedded (FFPE) tissue sections were used for spatial transcriptomics, which were performed according to the 10X Genomics Visium Spatial Gene Expression Reagent v2 Kit (10x Genomics, Pleasanton, CA, USA). Tissues were sectioned to 5 μm thickness and the slice was placed on the Visium Spatial Gene Expression Slide (10x Genomics) with barcoded spots. Next, paraffin was removed and the tissue was permeabilized to release mRNA. Reverse transcription was performed using the 10X Genomics Visium Spatial Tissue Optimization Kit, relying on oligo-dt primers seeded in 55 μm diameter capture spots on the Visium slide. Primers contain a spatial barcode to permit localization of sequence reads and unique molecular identifiers to exclude PCR-based duplicate reads. Lastly, the library was prepared using the 10X Genomics Spatial Gene Expression v2 Kit (10x Genomics). Sequencing was performed using an Illumina NextSeq (Illumina, San Diego, CA, USA).

### Serum aspartate aminotransferase (AST)

Serum aspartate aminotransferase (AST) activity was measured using a commercially available assay kit (NBP3-24470, Novus Biologicals, LLC, Centennial, CO, USA), according to manufacturer specifications.

### Serum alanine aminotransferase (ALT)

A commercially available assay kit (E-BC-K235-M, MSE Supplies LLC, Tucson, AZ, USA) was used to measure serum ALT activity, as we previously described [21].

### Serum major urinary protein 20 (MUP20)

A commercially available MUP20 ELISA kit (EKF58167, Biomatik, Ontario, Canada) was used to measure serum MUP20. Briefly, the ELISA plate was washed twice followed by being loaded with standards or samples. A biotin labeled antibody was added to each well, the plate was covered, and incubated at 37 °C for 45 min. Next, the plate was washed three times, an HRP-Streptavidin Conjugate was added to each well, and the plate was incubated at 37 °C for 30 min. Next, the plate was washed five times, a TMB substrate was added, and the plate was incubated at 37 °C for 20 min. Immediately following the incubation, stop solution was added and the absorbance of the plate was measured at 450 nm using an Agilent BioTek Synergy H1 plate reader.

### Western blotting

Western blotting was performed as previously described [21–24, 26]. Liver samples were put into RIPA buffer (89900, Thermo Fisher Scientific, Waltham, Massachusetts, USA) with added phosphatase (A32957, Thermo Fisher Scientific, Waltham, Massachusetts, USA) and protease (A32963, Thermo Fisher Scientific, Waltham, Massachusetts, USA) inhibitors and homogenized using a Teflon pestle. Homogenized samples were spun at 12,000 *g* for 10 min, the remaining supernatant was transferred to a new tube, and the total protein of the lysate was measured using a Pierce™ BCA Protein Assay Kit (23225, Thermo Fisher Scientific, Waltham, Massachusetts, USA). Between 20 and 30 µg of protein was separated by SDS-PAGE (Bio-Rad Laboratories, Hercules, CA, USA), transferred onto PVDF membranes, and the protein content in the membrane was determined using Ponceau S staining solution (40000279, Thermo Fisher Scientific, Waltham, Massachusetts, USA). The membranes were blocked in 5% TBST for one hour prior to being exposed to the primary antibody in 5% TBST overnight at 4 °C. The primary antibodies included fatty acid binding protein 5 (FABP5, 39926T, Cell Signaling Technologies, Danvers, MA, USA), glycogen phosphorylase brain isoform (PYGB, 12075-1-AP, Proteintech, Rosemont, IL, USA), and mechanistic target of rapamycin (mTOR, total mTOR antibody: 2983 S; mTOR phosphorylated at site serine 2448 antibody: 2971 S, both antibodies from Cell Signaling Technologies). After incubating with the primary antibody, the membranes were washed 5 times with TBST and then incubated with the anti-rabbit IgG, HRP-linked secondary antibody (7074, Cell Signaling Technologies) in 5% non-fat dry milk for 1 h at room temperature. Next, the membranes were washed 5 times with TBST, chemiluminescent substrate (34578, SuperSignal West Pico Plus, Thermo Fisher Scientific, Waltham, Massachusetts, USA) was added, and the membranes were imaged using an Odyssey Fc Imager (LI-COR Biosciences, Lincoln, NE, USA).

### Human liver biopsy samples acquisition

De-identified human liver tumor and matched normal tissue lysates were purchased from Novus Biologicals (Centennial, CO, USA). These samples were supplied as a 1 mg/ml lysate solution that was directly used in Western blotting. Since the human samples were collected elsewhere and de-identified, the Institutional Review Board at Pennington Biomedical Research Center determined that this is considered “Not human subjects research” as defined by 45 CFR Part 46.

### Proteomics

The proteomics experiment and follow-up bioinformatics analysis were performed at the IDEA National Resource for Quantitative Proteomics within the Winthrop P. Rockefeller Cancer Institute at the University of Arkansas. Samples were prepped and proteomics were performed as has been described elsewhere [27, 28].

### Promethion indirect calorimetry

The Promethion Core (Sable Systems International, Las Vegas, NV, USA) was used to measure oxygen consumption, respiratory exchange ratio, physical activity, and food intake. Approximately five days prior to metabolic testing, mice were initially placed in Promethion cages to acclimate mice to the new cage and living in isolation. Mice were tested for four consecutive days. On day three, mice were fasted for four hours prior to being injected intraperitoneally with 2 mg of total glucose (1.6 mg of ^12^C glucose and 0.4 mg of U^13^C glucose) per gram of body weight so that whole body glucose oxidation (^13^C carbon dioxide production) could be measured. Two hours after administering the glucose, mice were refed with their assigned diet.

### Statistics

Graphpad Prism 10.6.1 was used to perform some statistical analyses. A two-way ANOVA with follow-up Tukey’s comparisons were used to identify statistically significant effects (significance level was set at *P* ≤ 0.05) between groups. When only two groups were being compared, t-tests were performed. For oxygen consumption data, additional linear regression analyses were used to determine if body weight impacted absolute oxygen consumption values between groups, a statistical approach that is recommended for oxygen consumption data [29, 30]. The slope between the LFD and WD + DEN/TAA groups was not significantly different within males or females, indicating that any significant differences between oxygen consumption values between LFD and WD + DEN/TAA groups is not due to differences in body weights. Spatial transcriptomics were analyzed using Space Ranger to map reads to the spatial barcodes while Loupe Browser was used for visualization of the data. The spatial transcriptomics raw data was deposited into the Gene Expression Omnibus (GEO) data repository under series record GSE320362. ProteoDA was used to perform a statistical analysis on the proteomics data set. Proteins with an FDR adjusted P value < 0.05 and a fold change greater > 1.5 were considered significant. The Database for Annotation, Visualization, and Integrated Discovery (DAVID) was used to identify up-regulated or down-regulated cellular components and Kyoto Encyclopedia of Genes and Genomes (KEGG) pathways that were modified.

## Results

To establish a reliable method that induces advanced stage 2–3 HCC consistently in both male and female mice, we used the sequential administration of DEN, TAA, and 10% sucrose water while providing a solid Western diet from weaning age (Fig. 1A). The protocol was highly effective and produced many large visible liver tumors in both male and female mice, which is consistent with advanced HCC development (Fig. 1B, upper images). To further confirm the development of HCC, H&E was used and revealed clear cell carcinoma (large, irregularly shaped white (unstained) areas and a greater number of nuclei (blue stained areas) compared to LFD control mice (Fig. 1B). Additionally, Ki-67 staining, a marker of cell proliferation that is upregulated in HCC, was highly prevalent in both male and female mice with HCC but was nearly undetectable in liver sections from LFD control mice (Fig. 1B). Moreover, liver sections from mice in the WD + DEN/TAA group had greater fibrosis as indicated by positive staining for collagen using either Sirius Red / Fast Green stain (red stain is collagen, a marker of fibrosis) or Masson’s Trichrome stain (blue staining is collagen) (Fig. 1B). Final body weight was lower in LFD or WD + DEN/TAA female mice compared to male mice (Fig. 1C), with no impact of HCC on the final body weight within each sex. Consistent with this, there was no effect of HCC on fat (Fig. 1D) or lean mass (Fig. 1E), although female mice had lower lean mass compared to male mice (Fig. 1E). Liver weight was significantly elevated in both male and female WD + DEN/TAA groups compared to LFD control groups, indicating that large tumor mass increased the weight of the liver, an effect consistent with advanced HCC development (Fig. 1F). 100% of male (27/27) and female (29/29) mice had visible tumors > 2 mm in diameter, while mice in the LFD groups had no visible tumors (Fig. 1G). The total number of visible tumors was significantly greater in male mice compared to female mice (Fig. 1H). We next classified the mice into HCC stages using a scoring approach previously described that uses the number of macroscopic liver tumors with a diameter > 2 mm as an indicator of HCC stage [19]. Mice with no macroscopic liver tumors were considered stage 0, 1–3 tumors were considered stage 1 HCC, 4–6 tumors were considered stage 2 HCC, and 7 or more tumors were considered stage 3 HCC. Using this approach, 26 out of 27 male mice had stage 3 HCC, while 1 out of 27 had stage 2 HCC (Fig. 1I). In female mice, 19 out of 29 had stage 3 HCC, 9 out of 29 had stage 2 HCC, and 1 out of 29 had stage 1 HCC. Tumor weight varied and had a large range in both male and female mice but on average was lower in female mice compared to male mice (Fig. 1J). The majority of tumors that were weighed were between 1 and 100 mg for both sexes (Fig. 1K). Collectively, these data indicate this method reliably produces advanced stage 2–3 HCC in both male and female mice.

**Fig. 2:**
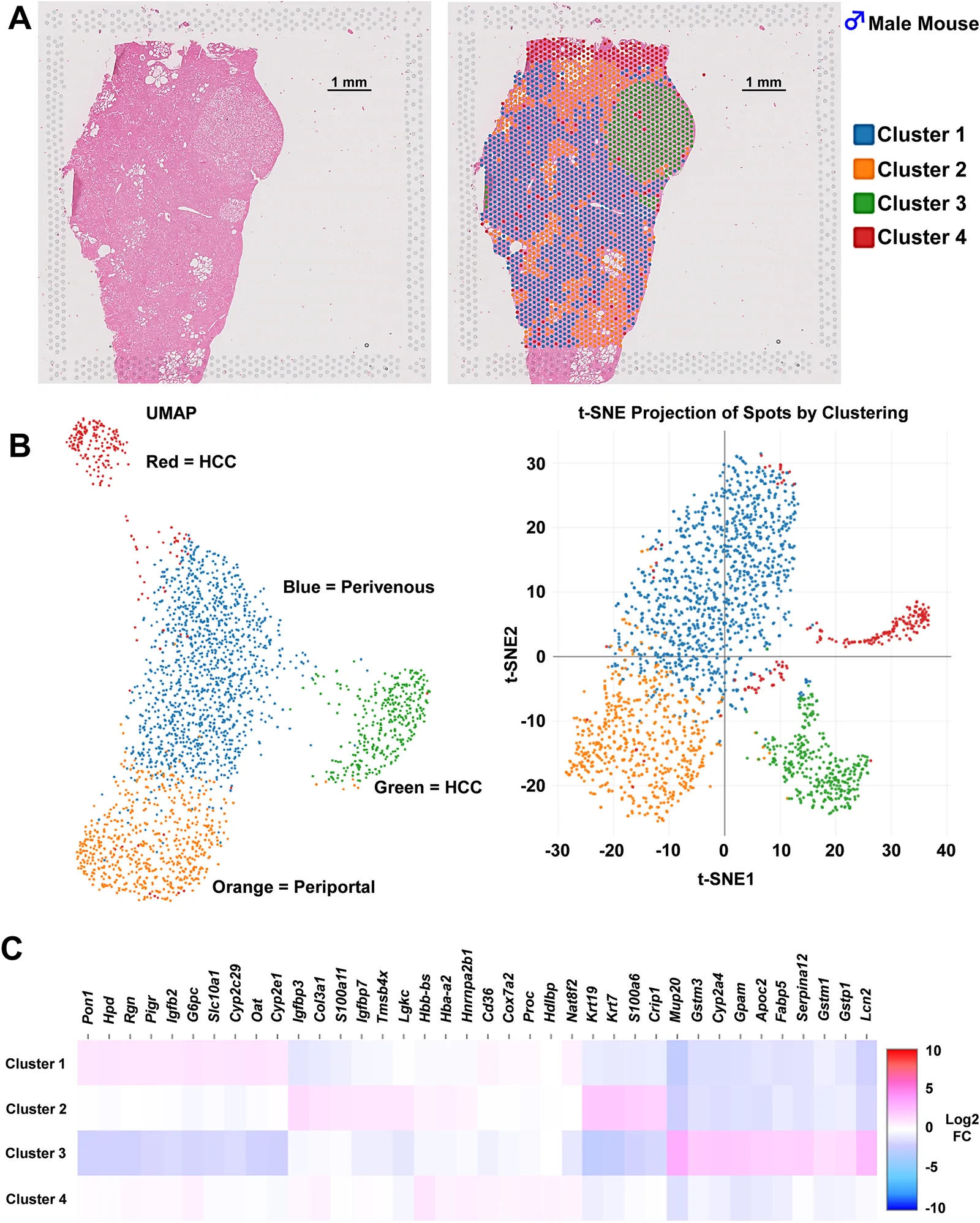
Spatial transcriptomics of male mouse HCC. **(A)** Eosin stained image within the capture area on the Visium Spatial Gene Expression Slide (left) and eosin stained image covered with barcoded spots (right) that were clustered based on their gene signatures. **(B)** A uniform manifold approximation and projection (UMAP) image visualizing the four gene clusters (left) and a t-distributed stochastic neighbor embedding (t-SNE) plot visualizing the four gene clusters identified in the liver tumor sample. **(C)** Heatmap of select genes that were differential expressed within each cluster (*n* = 1 male mouse liver section was used)

To further confirm that HCC developed and determine if this method induces HCC that has a similar gene expression profile compared to human HCC, we employed spatial transcriptomics using the 10X Genomics Visium platform. There were a total of 2,282 spots under the tissue, with means of 39,147 reads and 1,389 genes per spot. Eosin staining revealed normal liver tissue areas as well as several areas of HCC (Fig. 2A). The gene expression profile was classified into 4 clusters with unique gene signatures (Fig. 2A and B, Supplemental Table 1). Based on the morphological appearance of the tissue, cluster 1 and 2 are normal liver tissue areas. Cluster 1 (blue) had higher cytochrome P450 2e1 (*Cyp2e1*), ornithine aminotransferase (*Oat*), 6-phosphofructo-2-kinase/fructose-2,6-biphosphatase (*Pfkfb1*), and cytochrome P450 1a2 (*Cyp1a2*) cluster mean counts compared to cluster 2 (Supplemental Table 1), indicating these hepatocytes are likely perivenous hepatocytes as these genes have previously been validated to be more highly expressed within the perivenous zone [24, 31]. Cluster 2 (orange) had higher *Cdh1* cluster mean counts, which is a marker of periportal hepatocytes [24, 31], indicating this area is likely periportal hepatocytes. Cluster 3 (green) and cluster 4 (red) had gene signatures associated with HCC, albeit unique gene signatures highlighting that the tumor microenvironment can vary. Cluster 3 (green) and cluster 4 (red), compared to clusters 1 (blue) and 2 (orange), had higher abundance of markers known to be elevated in human HCC including fatty acid binding protein 5 (*Fabp5*) [32, 33] and lipocalin 2 (*Lcn2*) [34], indicating that the HCC produced from this method is similar to human HCC. Cluster 3 (green) had a higher cluster mean count of alpha fetoprotein (*Afp*), a marker of HCC [35], compared to cluster 4 (red), indicating that large tumors express *Afp*. Cluster 4 (red) had a high abundance of *Hdlbp*, a protein associated with HCC metastasis [36]. A heatmap of the top 10 differentially expressed genes between each cluster illustrates the unique gene signatures of the different HCC clusters (Fig. 2C). Overall, the method to induce HCC described here induced changes in gene expression that mirror some changes observed in human HCC [32–34, 37].

**Fig. 3:**
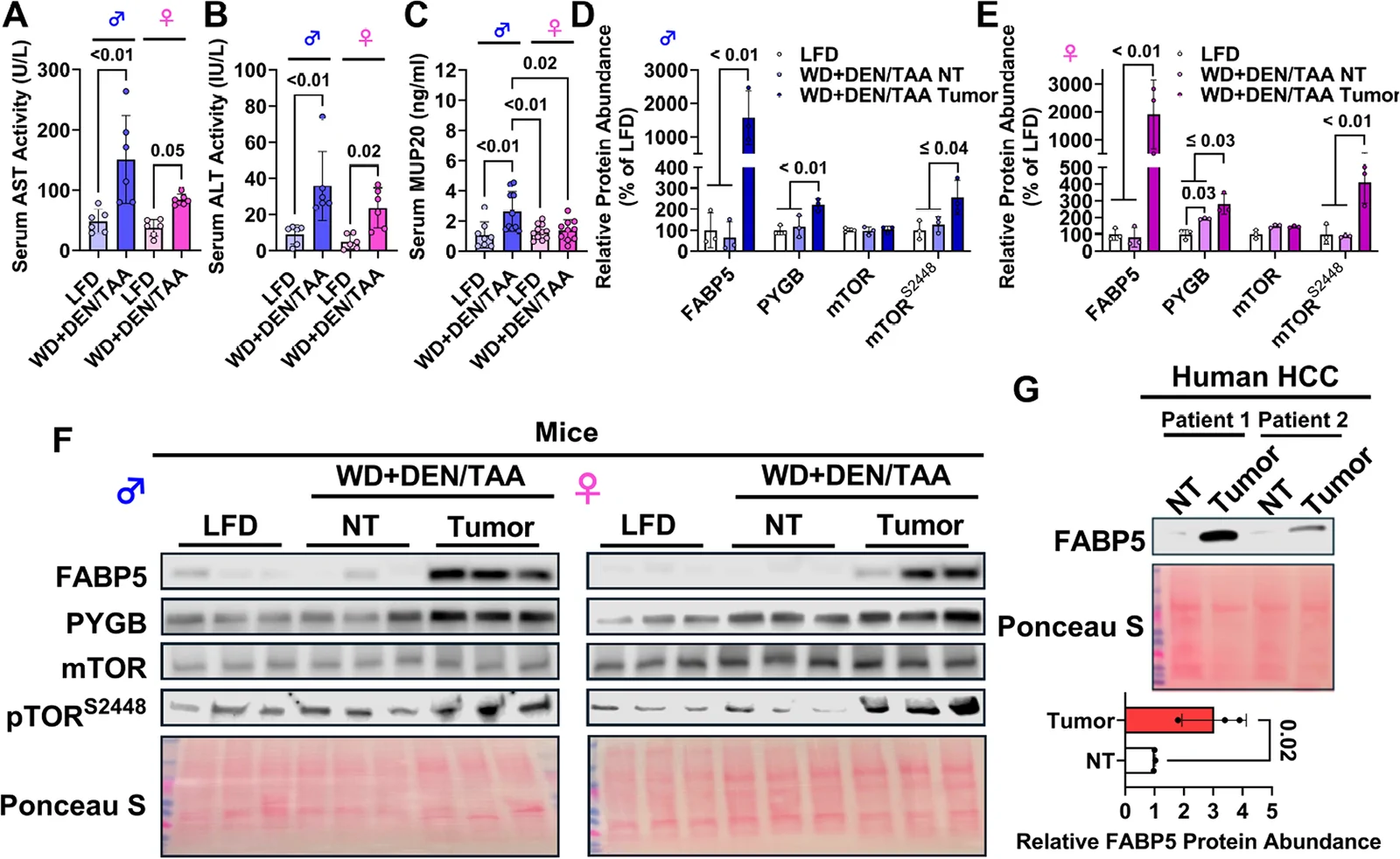
Markers of advanced HCC in serum and liver. **(A)** Serum AST activity in LFD and WD + DEN/TAA treated male and female mice (*n* = 6 for all groups). **(B)** Serum ALT activity in each group (*n* = 6 for all groups). **(C)** Serum MUP20 levels in each group (*n* = 8 for LFD fed males, *n* = 10 for WD + DEN/TAA males, *n* = 11 for LFD fed females, and *n* = 10 for WD + DEN/TAA females). **(D)** Densitometry quantification of protein abundance in LFD, WD + DEN/TAA non-tumor tissue (NT), and WD + DEN/TAA tumor tissue treated male mice, from blot images shown in F (*n* = 3 per group). **(E)** Densitometry quantification of protein abundance in LFD, WD + DEN/TAA NT, and WD + DEN/TAA tumor tissue treated female mice, from blot images shown in F (*n* = 3 per group). **(F)** Western blot images of mouse liver proteins typically upregulated in HCC (*n* = 3 per group). **(G)** Western blot images of human liver (NT and Tumor samples) proteins typically upregulated in HCC (*n* = 3 per group). Data are presented as means ± S.D

To further validate that this method induces advanced HCC, we also measured the protein abundance or activity of markers of HCC in the serum or liver tissue. Serum alanine aminotransferase (ALT) and aspartate aminotransferase (AST) are markers of liver damage and injury and are typically elevated in advanced HCC. Serum ALT and AST activity were both higher in the serum of WD + DEN/TAA treated male and female mice, compared to LFD male and female mice, providing evidence that advanced HCC has developed (Fig. 3A and B). The spatial transcriptomics data revealed an increase in *Mup20* gene abundance in the liver tumor from male mice. Consistent with this, male mice had higher MUP20 serum protein levels compared to LFD fed mice (Fig. 3C). However, LFD and WD + DEN/TAA female mice had similar serum MUP20 protein levels, indicating sex differences exist in this marker. Next, we performed western blotting on proteins well known to be elevated in human HCC including FABP5 [33], glycogen phosphorylase brain isoform (PYGB) [38], and mechanistic target of rapamycin (mTOR) [39]. These specific proteins were chosen as not only are they well established to be upregulated in human HCC, but validated antibodies are available to measure these markers. The protein abundance of FABP5 was significantly elevated in both mouse (Fig. 3D and F) and human (Fig. 3G) tumor tissue compared to adjacent, non-tumor tissue (NT) where its abundance was low and barely detectable. PYGB was also elevated in mouse tissue compared to adjacent, non-tumor tissue. We also observed an increase in phosphorylation of mTOR at serine 2448 (indicates higher mTOR activity) in mouse tumors compared to adjacent, non-tumor tissue and healthy tissue. Overall, these alterations in protein content are consistent with what has been observed in advanced human HCC [33, 38, 39].

**Fig. 4:**
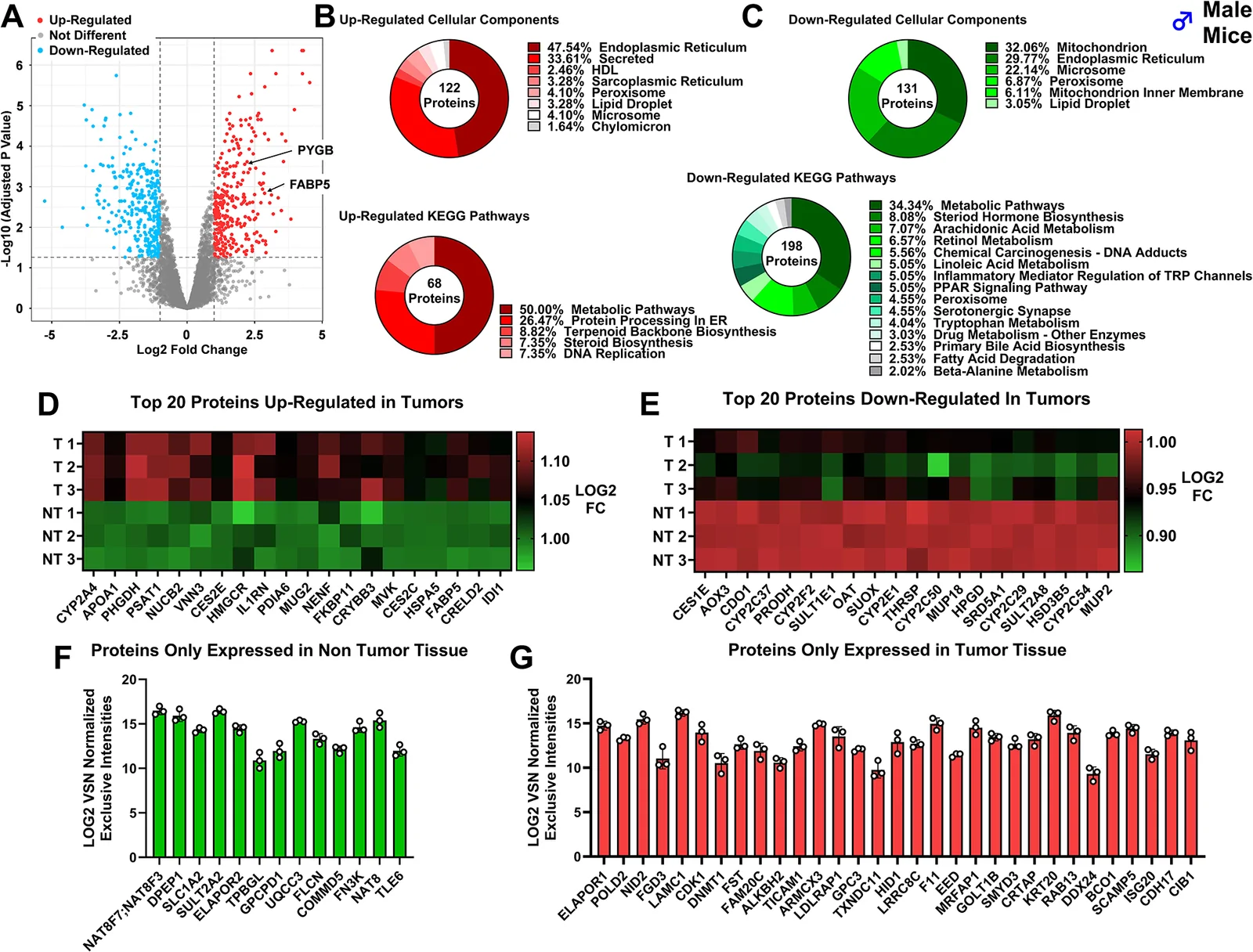
Proteomics of male mouse HCC. **(A)** Volcano plot illustrates significantly upregulated and downregulated proteins in tumors compared to adjacent, non-tumor tissue in WD + DEN/TAA treated mice (*n* = 3 total male mice, with *n* = 3 tumor (T) samples and *n* = 3 non-tumor (NT) samples per group, while the T and NT tissue was paired and taken from the same mouse). **(B)** Select up-regulated cellular components and KEGG pathways in HCC T compared to NT. **(C)** Select down-regulated cellular components or KEGG pathways in HCC T compared to NT. **(D)** Heatmap of top 20 up-regulated proteins in HCC T compared to NT. **(E)** Heatmap of top 20 down-regulated proteins in HCC T compared to NT. **(F)** Proteins that were only expressed in NT tissue. **(G)** Proteins that were only expressed in HCC T

Next, a proteomics analysis of tumor and adjacent, non-tumor tissue in WD + DEN/TAA treated male mice was performed. A volcano plot revealed a total of 153 proteins were up-regulated and 161 proteins were down-regulated in tumor tissue compared to adjacent, non-tumor tissue (Fig. 4A, Supplemental Table 2). FABP5 and PYGB were both up-regulated in the proteomics analysis, which is consistent with our western blotting data. Next, the DAVID Bioinformatics database was used to further characterize the proteome in this model. Functional annotation analysis revealed that the majority of up-regulated proteins were classified as endoplasmic reticulum or secreted cellular components (Fig. 4B, upper figure), while the majority of down-regulated proteins were in the mitochondrion, endoplasmic reticulum, or microsome (Fig. 4C, upper figure). A KEGG pathway analysis revealed that the majority of up-regulated proteins were within metabolic pathways or protein processing in the endoplasmic reticulum (Fig. 4B, lower figure), while the majority of down regulated proteins were within metabolic pathways, steroid hormone biosynthesis, or arachidonic acid metabolism (Fig. 4C, lower figure). A heatmap of the top 20 proteins (based on fold change) that were increased revealed FABP5 was elevated as well as proteins involved in terpenoid backbone biosynthesis including HMGCR, IDI1, and MVK (Fig. 4D). A heatmap of the top 20 proteins (based on fold change) that were decreased revealed several proteins involved in steroid hormone biosynthesis and arachidonic acid metabolism including CYP2C29, CYP2C50, CYP2C54, CYP2E1, CYP2C37, HSD3B5, SRD5A1, SULT1E1, and HPGD (Fig. 4E). There were also 13 proteins that were only expressed in non-tumor tissue and not in tumor tissues, with three of these proteins being involved in glutathione metabolism including NAT8FS, NAT8F7, and NAT8 (Fig. 4F). Conversely, 31 proteins were uniquely expressed in tumor-tissue, but not non-tumor tissue, with several proteins involved in chromatin regulation including DNMT1, SMYD3, and EED (Fig. 4G). Moreover, glypican 3 (GPC3) was found only in tumors and is a known maker for advanced HCC [40, 41], indicating that our method is inducing advanced HCC.

**Fig. 5:**
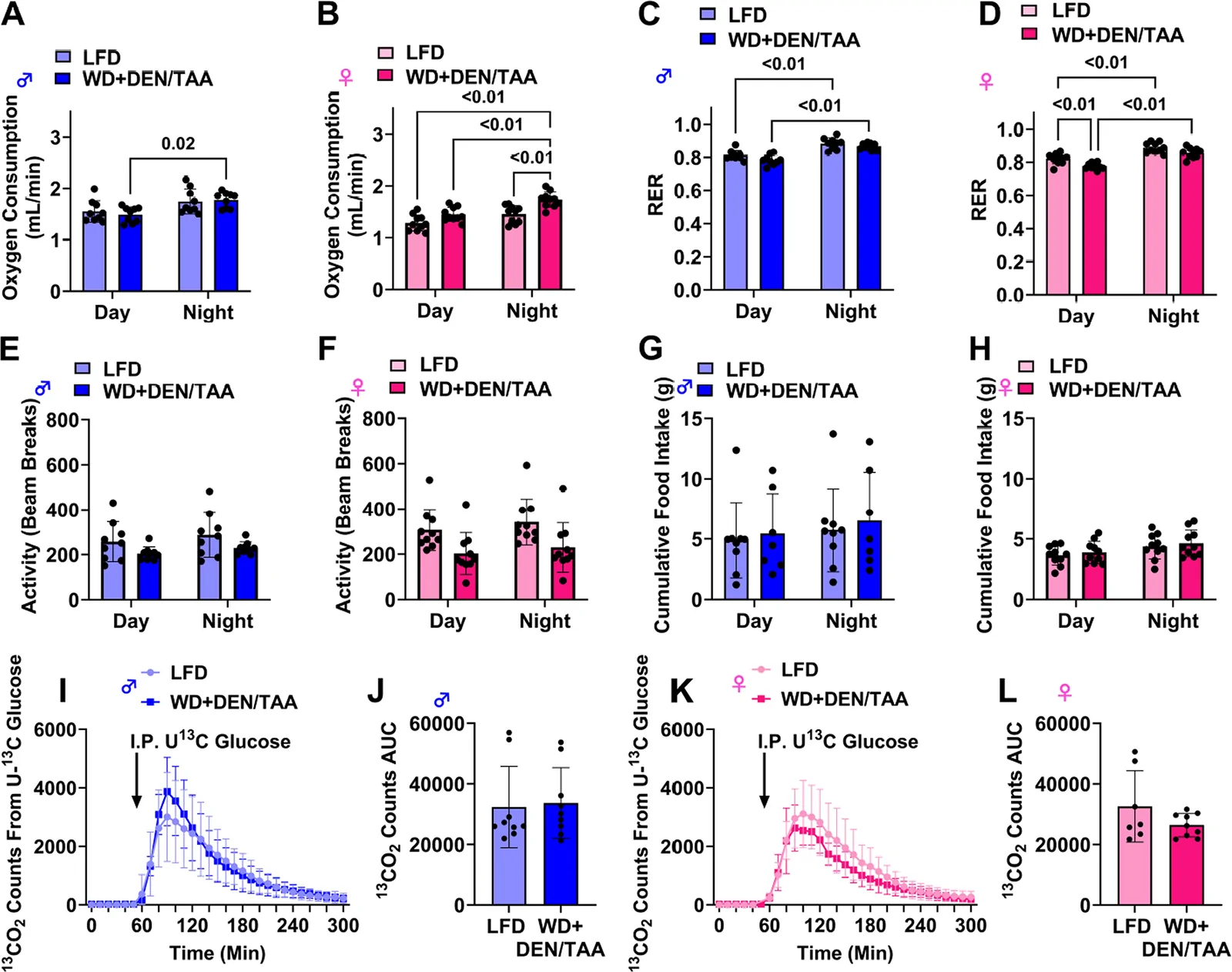
Whole body metabolism, physical activity, and food intake. **(A-B)** Average oxygen consumption rates during the day and night in male and female mice (*n* = 9 LFD fed males, *n* = 9 WD + DEN/TAA males, *n* = 10 LFD fed females, and *n* = 10 WD + DEN/TAA females). **(C-D)** Average RER during the day and night (*n* = 9 LFD fed males, *n* = 9 WD + DEN/TAA males, *n* = 10 LFD fed females, and *n* = 10 WD + DEN/TAA females). **(E-F)** Average physical activity levels (beam breaks) (*n* = 9 LFD fed males, *n* = 9 WD + DEN/TAA males, *n* = 10 LFD fed females, and *n* = 10 WD + DEN/TAA females). **(G-H)** Cumulative food intake during the day and night (*n* = 9 LFD fed males, *n* = 7 WD + DEN/TAA males, *n* = 10 LFD fed females, and *n* = 10 WD + DEN/TAA females). **(I-L)** Whole body glucose oxidation measured as amount of ^13^CO2 in exhaled air (*n* = 9 LFD fed males, *n* = 9 WD + DEN/TAA males, *n* = 7 LFD fed females, and *n* = 9 WD + DEN/TAA females). Data are presented as means ± S.D

Some studies suggest that resting energy expenditure measured with indirect calorimetry is higher in humans with HCC compared to those without HCC [42, 43]. Next, the Promethion high-resolution indirect calorimetry system was used to determine if our protocol alters whole body energy metabolism in mice. In male mice, oxygen consumption was higher at night compared to the day in the WD + DEN/TAA treated group, whereas no other significant differences existed between groups (Fig. 5A). In female mice, oxygen consumption was highest in the WD + DEN/TAA group at night compared to the WD + DEN/TAA group during the day or compared to the LFD group during the day or night (Fig. 5B). In male mice, the respiratory exchange ratio (RER) was higher during the night compared to the day, regardless of group (Fig. 5C). In female mice and during the day, the RER was lower in the WD + DEN/TAA group compared to the LFD group (Fig. 5D). During the night, these differences in RER between groups disappeared. Moreover, the RER was greater at night in both LFD and WD + DEN/TAA groups compared to the day. These differences in oxygen consumption and substrate metabolism were not associated with changes in physical activity levels (Fig. 5E and F) or food intake (Fig. 5G and H). Moreover, whole body complete glucose oxidation to CO_2_ after an I.P. injection of U^13^C glucose was not different between the groups (Fig. 5I and L).

## Discussion

Preclinical mouse models that closely mimic the human disease are a foundational element of translational science and are required to better understand the pathogenesis of the disease as well as identify and test the efficacy and safety of new therapies prior to initiating human clinical trials. DEN is a very common genotoxic compound used to promote HCC in preclinical mouse models, yet its use results in inconsistent HCC occurrence, particularly in female mice [4–9, 11–14]. In this study, we resolved this issue and report that eight DEN injections spaced one week apart and starting at two weeks of age (from age 2–9), followed by TAA administration for four weeks (from age 11–14), and then 10% sucrose water (from age 17–30), all on top of a solid Western diet, consistently and reliably promote advanced stage 2–3 HCC in both male and female mice by 30 weeks of age. Importantly, this method altered serum or liver HCC markers in a direction that is consistent with advanced human HCC, making this preclinical mouse model highly translational. This knowledge will be useful for future studies examining the pathogenesis of HCC or testing new therapeutic approaches for advanced HCC.

Lipid metabolism undergoes a significant remodeling in the liver cancer microenvironment, moving toward a state of increased lipid synthesis, uptake, and storage to fuel rapid cell growth, proliferation, and survival in the stressful tumor microenvironment [44–47]. Our spatial transcriptomics analysis in conjunction with proteomics and western blotting revealed that FABP5, a lipid chaperone protein involved in lipid trafficking and membrane synthesis, was upregulated in tumors compared to adjacent, non-tumor tissue in our mouse model, and this effect mirrored what was observed in our human HCC samples. Previous studies have shown that FABP5 is upregulated in human advanced HCC and associated with poor prognosis and lower survival rates [33, 48, 49]. In cell culture, upregulating FABP5 increases proliferation [33, 48], while knockdown of FABP5 reduces proliferation in various HCC cell lines [33]. Moreover, in mice genetic ablation of FABP5 reduced HCC burden [47]. Taken together, FABP5 is a physiological marker of human HCC that is also upregulated in our preclinical mouse model.

Lipocalin-2 (LCN2) is a secreted glycoprotein and iron-sequestering cytokine that is upregulated in human HCC and can be used as a diagnostic marker [34, 50]. LCN2 promotes glycolysis and proliferation through the JAK2/STAT3 pathway [34]. Another report indicated that loss of leukemia inhibitory factor receptor in HCC promotes nuclear factor kappa-light-chain-enhancer of activated B cells signaling which upregulates LCN2 to deplete iron and reduce ferroptosis [51]. Thus, LCN2 is a key player in HCC progression. Our spatial transcriptomics data showed that *Lcn2* mRNA abundance was highly upregulated in tumors compared to non-tumor tissue, while our proteomic data showed that LCN2 protein abundance was also higher in tumor tissue compared to non-tumor tissue. Given that LCN2 protein was upregulated in this mouse model indicates the protocol is able to induce a key feature observed in human HCC.

Glycogen phosphorylase brain isoform (PYGB) is one of three isoforms of glycogen phosphorylase and facilitates glycogenolysis to form glucose 1-phosphate or glucosamine 1-phosphate [52]. Although initially found to be highly expressed in brain tissue, its expression is high in other tissues and various cancers [51], including HCC [38, 49]. In HCC, PYGB is a better predictor of poor prognosis compared to glycogen phosphorylase liver isoform (PYGL) [38], while its inhibition reduces cell proliferation in HCC cell models [38, 53]. Although localized to the cytoplasm, PYGB is also found in the nucleus where it promotes glycogenolysis to provide substrates for histone acetylation [51]. The mouse model presented herein upregulates PYGB similar to human HCC, indicating that our model recapitulates a key feature of human HCC.

mTOR is an atypical serine/threonine kinase and a master growth regulator that is recruited to the lysosome prior to activation. mTOR responds to a diverse range of extracellular and intracellular cues to control cell growth and limit catabolism [54]. mTOR signaling is upregulated in human HCC [55, 56], while in a mouse model overactivation of mTOR is sufficient to drive HCC development [57]. Consistent with this, mTOR signaling was elevated in our mouse model. These findings indicate that our model will be suitable to test how mTOR inhibitors impact HCC.

A unique feature of this model is that it promoted advanced HCC in female mice that are normally protected by estrogen and have a very low incidence of HCC [11]. However, a weakness of this research is that female samples were not used in the spatial transcriptomics or proteomics data sets. As a result, the molecular characterization and generalizability of this model to female mice is limited, and the mechanisms by which this model promotes advanced HCC in female mice is not clear. We speculate that the potential mechanism for this effect may be that the sequential administration of DEN, TAA, and sucrose water provided herein provides enough insults to overwhelm the ability of estrogen to protect the liver from HCC in female mice. However, more research is needed to identify an exact mechanism and signaling pathways. On average, female mice did have a lower number of visible tumors, mice in stage 3, and lower tumor weight compared to male counterparts, indicating they still have some mild protection in this model.

## Conclusion

In conclusion, this study introduces, for the first time, a method that utilizes the sequential administration of chemical carcinogens and sucrose water, in conjunction with a solid Western diet from weaning, to reliably induce advanced HCC in both male and female mice. Importantly, the model was highly tolerable and induced some protein and gene signatures that are comparable to what has been observed in human HCC. This new method to induce HCC will be a valuable preclinical model for better understanding the pathogenesis of HCC to the advanced stage or preclinical testing of new therapeutic approaches for advanced stage HCC.

## Supplementary Information

Below is the link to the electronic supplementary material.

Supplementary Material 1

Supplementary Material 2

Supplementary Material 3

## Data Availability

The datasets used and analyzed during the current study are available from the corresponding author on reasonable request. The spatial transcriptomics raw data was deposited into the Gene Expression Omnibus (GEO) data repository under series record GSE320362.

## Abbreviations

ALT: Alanine aminotransferase
AST: Aspartate aminotransferase
DEN: Diethylnitrosamine
DNA: Deoxyribonucleic acid
FABP5: Fatty acid binding protein 5
FFPE: Formalin fixed paraffin embedded
HCC: Hepatocellular carcinoma
mTOR: Mechanistic target of rapamycin
MUP20: Major urinary protein 20
LFD: Low-fat diet
PYGB: Glycogen phosphorylase brain isoform
TAA: Thioacetamide
WD: Western diet
