# Supplementary material for: A method utilizing chemical carcinogens and a Western diet to reliably induce advanced hepatocellular carcinoma in male and female mice
Source: Cancer Metab. 2026 Jun 9;14:32. doi: 10.1186/s40170-026-00441-6 (PMC13660167; doi:10.1186/s40170-026-00441-6)

Figure 3F Uncropped Western Blot Images - Males

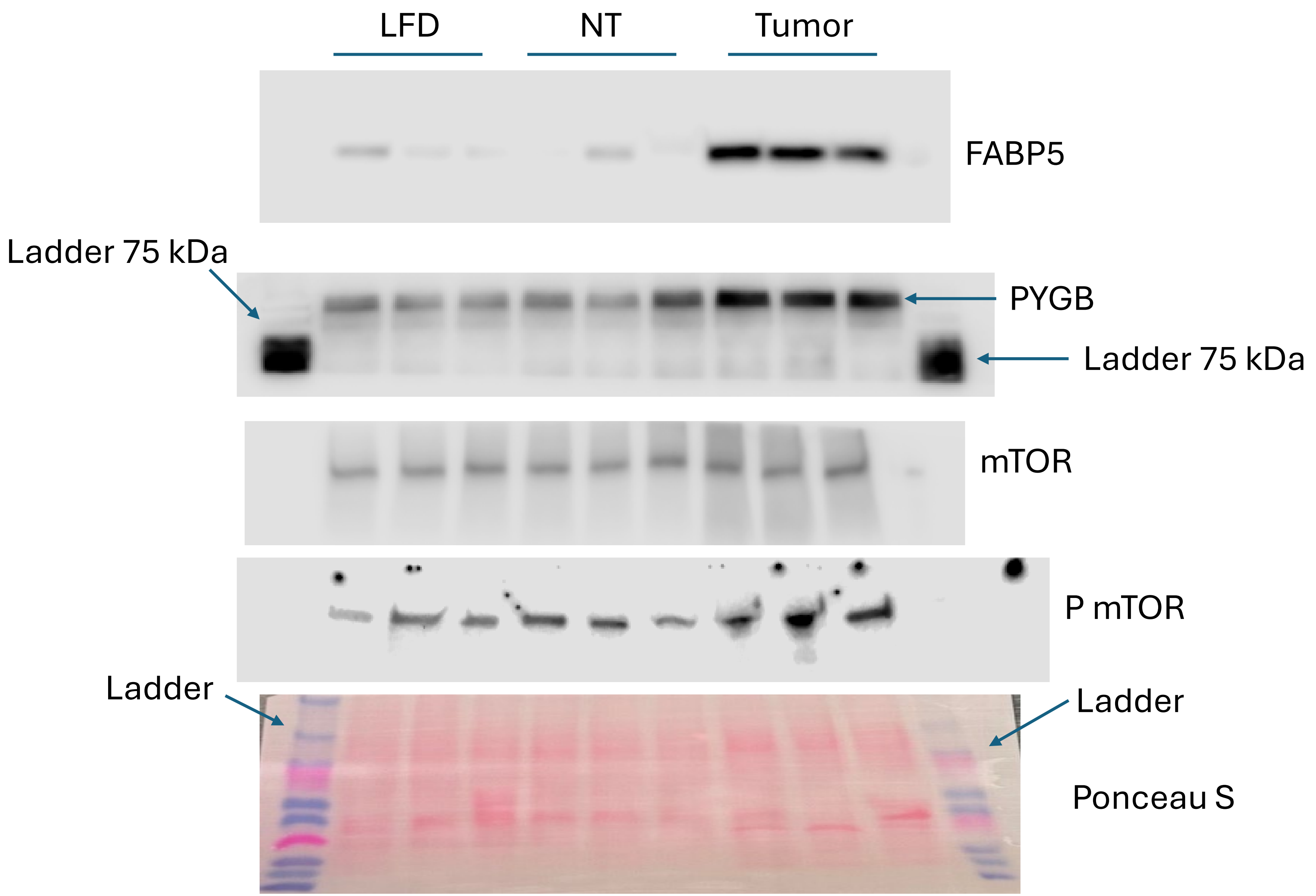

Figure 3F Uncropped Western Blot Images - Females

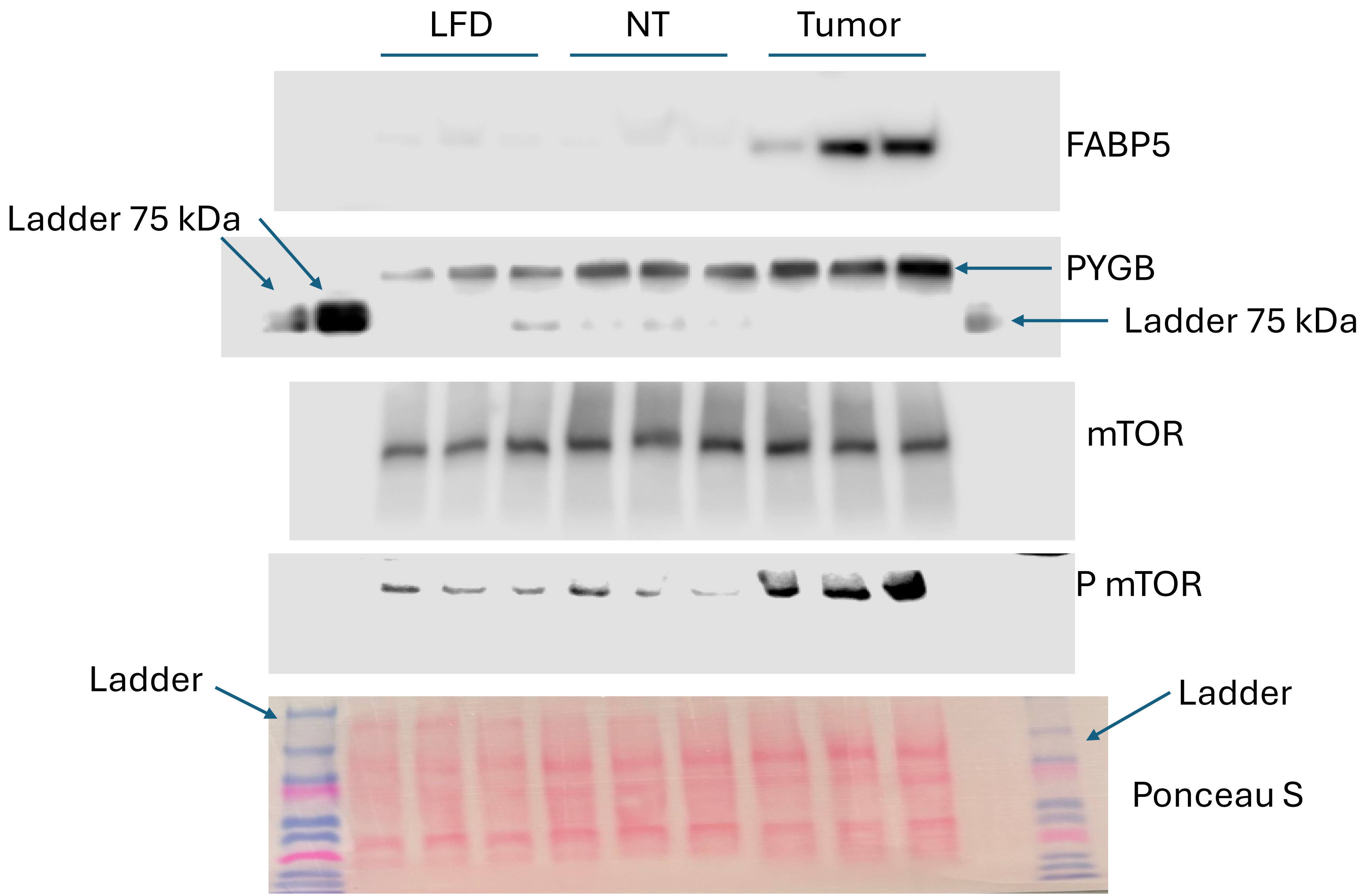

Figure 3G Uncropped Western Blot Images - Human

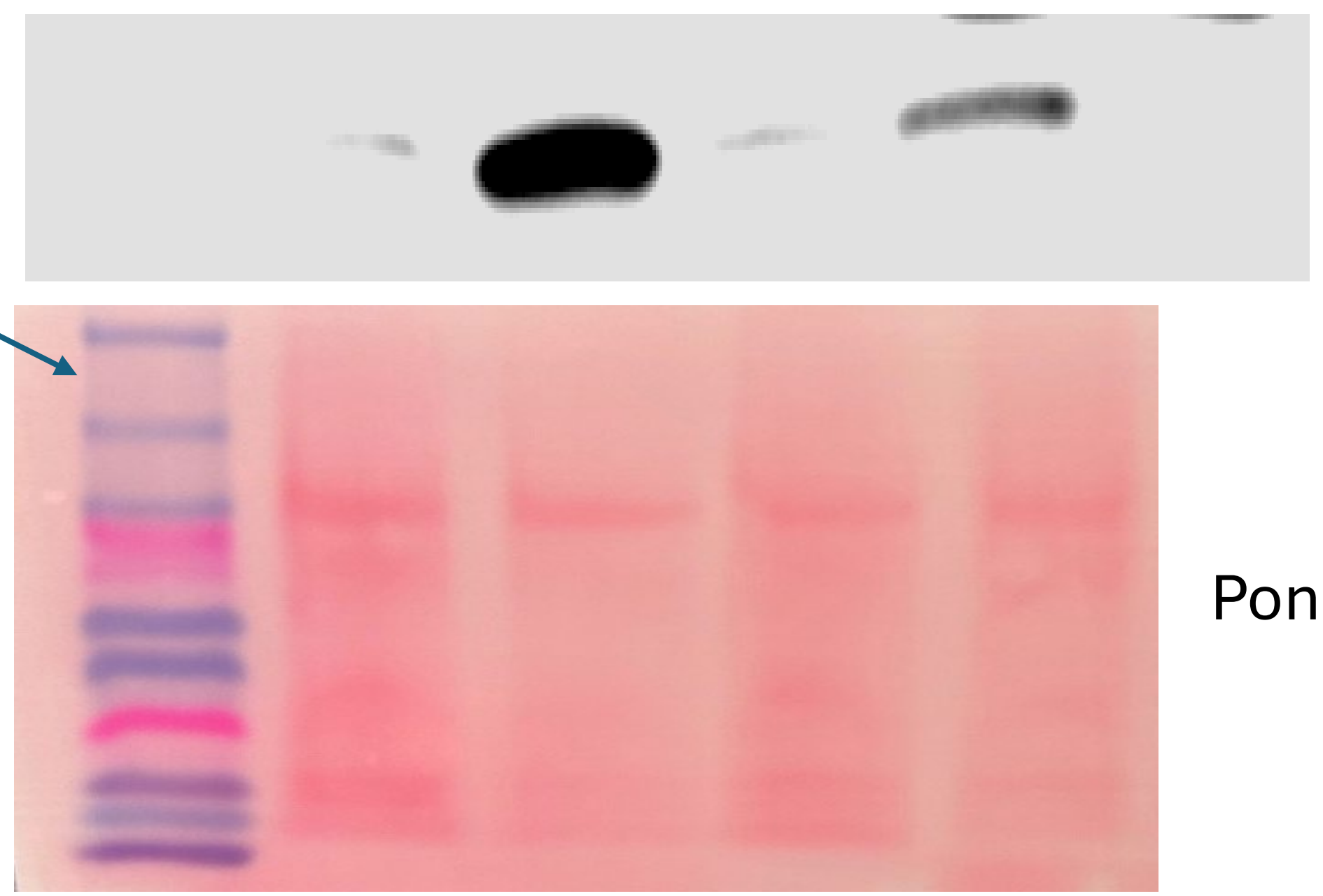

Supplement: Supplementary file 1 [file 40170_2026_441_MOESM3_ESM.pdf]
